# Flexible gold standards for transcription factor regulatory interactions in *Escherichia coli* K-12: architecture of evidence types

**DOI:** 10.3389/fgene.2024.1353553

**Published:** 2024-03-05

**Authors:** Paloma Lara, Socorro Gama-Castro, Heladia Salgado, Claire Rioualen, Víctor H. Tierrafría, Luis J. Muñiz-Rascado, César Bonavides-Martínez, Julio Collado-Vides

**Affiliations:** ^1^ Centro de Ciencias Genómicas, Universidad Nacional Autónoma de México, Avenida Universidad S/N, Cuernavaca, Mexico; ^2^ Department of Biomedical Engineering, Boston University, Boston, MA, United States; ^3^ Center for Genomic Regulation, The Barcelona Institute of Science and Technology, Universitat Pompeu Fabra, Barcelona, Spain

**Keywords:** regulatory interactions, *E. coli*, evidence codes, source of knowledge, confidence levels, high-throughput genomic methodologies, RegulonDB

## Abstract

Post-genomic implementations have expanded the experimental strategies to identify elements involved in the regulation of transcription initiation. Here, we present for the first time a detailed analysis of the sources of knowledge supporting the collection of transcriptional regulatory interactions (RIs) of *Escherichia coli* K-12. An RI groups the transcription factor, its effect (positive or negative) and the regulated target, a promoter, a gene or transcription unit. We improved the evidence codes so that specific methods are incorporated and classified into independent groups. On this basis we updated the computation of confidence levels, weak, strong, or confirmed, for the collection of RIs. These updates enabled us to map the RI set to the current collection of HT TF-binding datasets from ChIP-seq, ChIP-exo, gSELEX and DAP-seq in RegulonDB, enriching in this way the evidence of close to one-quarter (1329) of RIs from the current total 5446 RIs. Based on the new computational capabilities of our improved annotation of evidence sources, we can now analyze the internal architecture of evidence, their categories (experimental, classical, HT, computational), and confidence levels. This is how we know that the joint contribution of HT and computational methods increase the overall fraction of reliable RIs (the sum of confirmed and strong evidence) from 49% to 71%. Thus, the current collection has 3912 reliable RIs, with 2718 or 70% of them with classical evidence which can be used to benchmark novel HT methods. Users can selectively exclude the method they want to benchmark, or keep for instance only the confirmed interactions. The recovery of regulatory sites in RegulonDB by the different HT methods ranges between 33% by ChIP-exo to 76% by ChIP-seq although as discussed, many potential confounding factors limit their interpretation. The collection of improvements reported here provides a solid foundation to incorporate new methods and data, and to further integrate the diverse sources of knowledge of the different components of the transcriptional regulatory network. There is no other genomic database that offers this comprehensive high-quality architecture of knowledge supporting a corpus of transcriptional regulatory interactions.

## 1 Introduction

Genomic sciences have expanded the landscape of available experimental strategies to identify, on a genomic scale, a variety of genetic elements, such as transcription factor binding sites (TFBSs) and their subset of transcription factor regulatory sites (TFRSs), i.e., those TFBSs with regulatory evidence for a given transcription factor (TF); transcription start sites (TSSs), transcription termination sites (TTSs), as well as transcription units (TUs), all of these in principle under defined growth conditions.

We have been for the last 30 years continuously extracting and gathering in RegulonDB and feeding into EcoCyc knowledge supported by classic molecular biology methods from original scientific publications about regulation of transcription initiation and operon organization in *Escherichia coli* K-12. Although we have for years curated HT data, only recently, since RegulonDB version 11.0, we incorporated collections of publicly available genomic HT datasets of binding sites (from chromatin immunoprecipitation combined with sequencing (ChIP-seq), ChIP combined with exonuclease digestion and next-generation sequencing (ChIP-exo), genomic SELEX screening (gSELEX), and DNA affinity purification sequencing (DAP-seq) technologies), of TSSs, TTSs, TUs, and normalized RNA-seq expression profiles (Tierrafría et al., 2022). This included the update of some datasets so that all datasets are uniformly described in a single format, and all coordinates are in the current genome version enabling their comparability. In our curation work, we have seen that the publications of these types of approaches frequently compare the obtained results with what is known in RegulonDB (Shimada et al., 2011a; Shimada et al., 2011b; Shimada et al., 2011c; Kahramanoglou et al., 2011; Seo et al., 2014; Shimada et al., 2015a; Shimada et al., 2015b; Ishihama et al., 2016; Kim et al., 2018; Shimada et al., 2018; Kroner et al., 2019; Anzai et al., 2020; Choudhary et al., 2020; Baumgart et al., 2021; Ishihama and Shimada, 2021; Shimada et al., 2021). This motivated us to improve our evidence codes to enhance the use of RegulonDB as “gold standard”. Certainly, evidence codes used for years both in RegulonDB and EcoCyc were not detailed enough to distinguish different methods. For instance, the terms “binding of purified proteins” or “gene expression analysis” did not specify the method.

The need to easily distinguish objects based on the approach used (i.e., classic vs. HT methods), the fact that RegulonDB sites are used as an index to evaluate the performance of novel methods, and the desire to enhance the precision to access the literature behind specific properties of complex objects such as regulatory interactions (RIs) or promoters, motivated us to update the evidence codes behind the knowledge on the regulation of transcription initiation. The new codes distinguish not only the class of methods but also the specific methodology, for instance ChIP-seq, ChIP-exo or gSELEX. We began moving in this direction a few years ago, but only now we integrate and report a series of changes that improve knowledge representation in RegulonDB, enabling analyses as shown below.

Once the new evidence types were defined and groups of independent methods were redefined, we reconstructed the way in which combinatorial rules combine to determine the “confidence level” based on the set of evidence types behind an object, assigning it as either weak, strong, or confirmed. We have mapped the existing RIs with the HT-TFBSs collections and added the corresponding HT binding evidence types to the RIs, improving their confidence level. This was updated in the RegulonDB 12.2 version.

Additionally, we implemented three levels of representation of regulatory interactions to adequately deal with cases with partial knowledge, as explained below. All these modifications contribute to a significant improvement in the quality of knowledge representation in RegulonDB. This allows us to perform what we believe is the first highly detailed analysis of the sources of knowledge supporting the current transcriptional regulatory network of a genome, the one of *E. coli* K-12. The analysis of the architecture, or anatomy, of this corpus of knowledge enables us to quantify, for instance, the increasing contribution of HT methods and their effect in the distribution of highly confident interactions, the dominant classical methods behind these interactions, as well as a preliminary comparison of the recovery of classical TFRSs by the different HT methods.

Furthermore, taken together, all these improvements constitute a solid and flexible foundation towards the major challenge of constructing the most up to transcriptional regulatory network with a high-quality documentation of its sources of knowledge.

## 2 Results

A regulatory interaction is one of the major concepts, together with transcription units and operons, of gene regulation and specifically of regulation of transcription initiation. As with any piece of knowledge, it can be described at different levels of detail; at a low level we can say it is the triplet formed by the TF, the target gene, and the positive or negative effect. The basic requisites to annotate a new RI are: evidence of a TF binding near the gene start and the functional evidence showing that the presence/absence of this TF has a positive or negative effect on the target gene. In contrast, at a high level of detail, knowledge of RIs involves the TF, the effector that affects its binding and unbinding conformation, the precise TF regulatory binding site, the regulated promoter, and the effect of the TF when bound, either activating or repressing transcription initiation. It may also be expanded and include knowledge of the TU that the promoter transcribes and therefore the set of regulated genes, and finally the growing conditions (experimental and control) under which such regulation has been shown.

Following an update of basic concepts of transcriptional regulation (Mejía-Almonte et al., 2020), we implemented some modifications, both in RegulonDB and EcoCyc, to conform to the new definitions. One that is relevant to this work is the distinction between TF binding sites (TFBSs) and the subset of TF regulatory sites (TFRSs), which are those TFBSs with functional evidence showing they have a regulatory role. This distinction is particularly useful as genomic methods identify TFBSs; but only a few of them have evidence of their regulatory role on target genes or promoters. TFBSs *per se* do not support RIs.

### 2.1 Updated and new evidence codes

We updated our table of evidence types, and have modified their descriptions to explicitly include whether they are experimental methods, either HT or classic methods, or nonexperimental, such as computational predictions or author statements. We modified the names of evidence codes to make them more informative. Since some objects are rather complex, particularly the RIs, we have separated the evidence for binding within sites and the evidence for function within the RI itself. This also facilitates user searches for specific references for different properties of complex objects. Each evidence type is associated with a specific code, as short as possible but informative and with prefixes indicating if it is an HT method.

We added a link in RegulonDB that offers the name, description, evidence code, and confidence level (see below) of all evidence types, as well as whether they correspond to *in vitro* or *in vivo* binding experiments. **See:** https://regulondb.ccg.unam.mx/manual/help/evidenceclassification **.** Although this table shows updated codes for RIs, promoters, and TUs, in this paper we only focus on RIs. As can be seen, the new evidence types added are essentially those that support HT methods.

### 2.2 Confidence derived from multiple independent methods

Years ago, we classified in RegulonDB the different evidence types into either weak or strong, based on the confidence that the methods provided to support the existence of a piece of knowledge. The general principle is that strong confidence comes from experiments that provide clear physical evidence of the existence of the object. For instance, binding of purified proteins is considered strong evidence, whereas binding of cellular extracts is considered weak evidence. A limitation to this initial approach was that even if some objects are identified by different methods, either in the same paper or through the years in more than one publication, we did not have a process to add multiple weak evidence types and upgrade the confidence of the object. This is contrary to a fundamental practice in natural science, whereby further support to knowledge is gained by different, and ideally independent strategies or methods. We analyzed which of the different methods can be considered independent because they use different assumptions and/or different methodological strategies and their potential sources of error are different (Weiss et al., 2013). It is on this basis that we built our algebra to combine multiple weak independent sources of methods into a strong confidence level. We also proposed the combination of independent strong evidence types to create the new “confirmed” level of confidence.

In the current update, we kept the same principles and criteria as defined in the 2013 paper (Weiss et al., 2013) and made a series of changes that will become clear as they are required to perform the analyses of the sources of knowledge presented in this paper. These improvements are explicitly summarized in the discussion.

**FIGURE 1:**
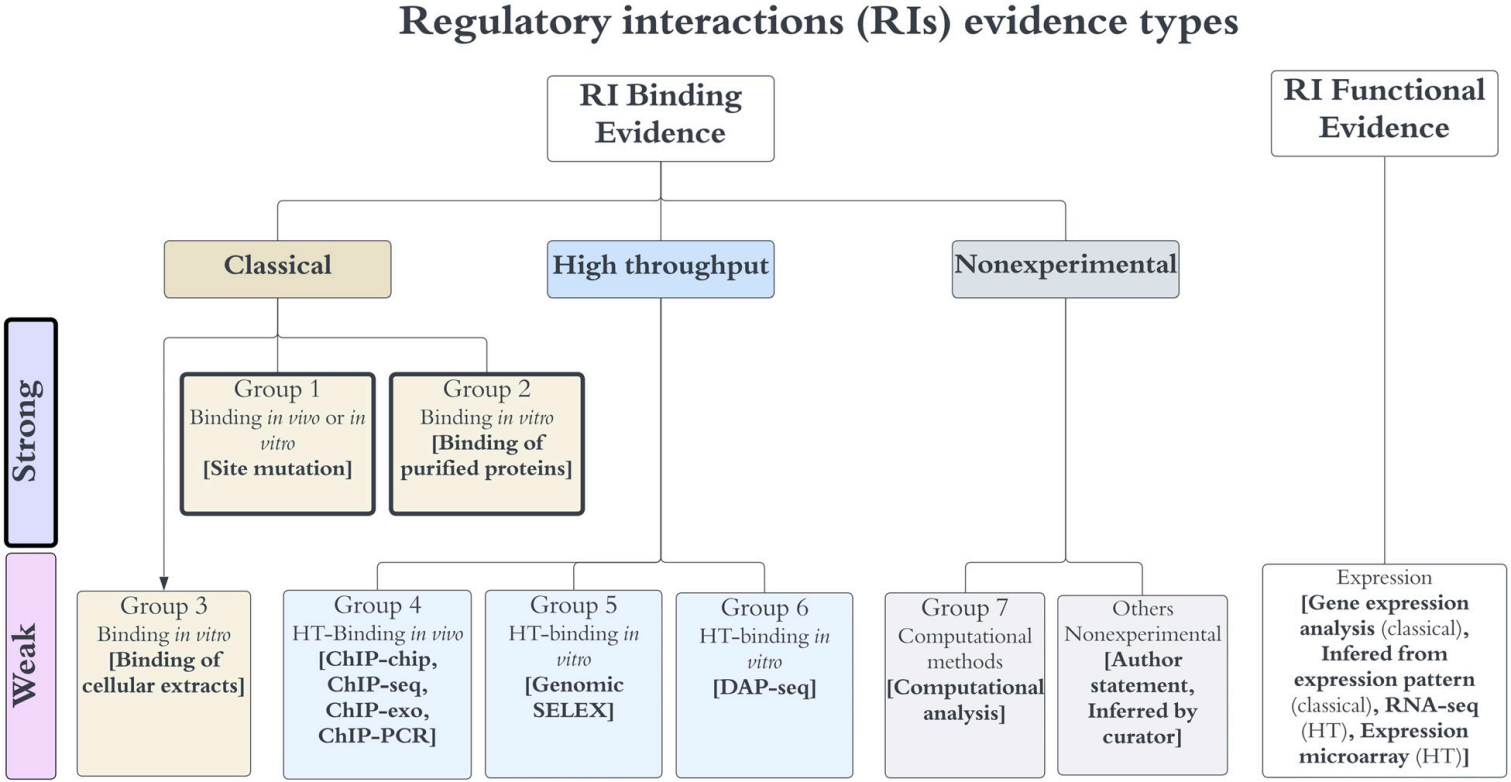
Evidence types for RIs. Any RI requires evidence for the binding of the TF, together with functional evidence showing its regulatory effect in transcriptional activity. Evidence types are grouped in three major categories (classical, HT and nonexperimental), each specific group contains methods that are not considered independent, whereas methods of different groups are considered independent. The algebra of their independent groupings is limited to binding evidence types, which define the level of confidence as discussed in the main text. Note that the “others nonexperimental” evidence is not used to assign confidence.

In the case of classical evidence, most methods provide direct physical evidence, so they are classified as strong, except the binding of cellular extracts, since such experiments do not eliminate the possibility of indirect effects. HT binding evidence types were classified as weak, since they involve several processing steps, including different bioinformatics options of methods and thresholds, making the final results more variable and dependent on the specific set of programs and variables used in their final identification. Thus, processing the same raw data may potentially result in different final collections of objects; in addition, there is no consensus yet on a uniform processing pipeline used by the community. Nonexperimental evidence types were also classified as weak; however, among them only computational analysis can be used in combination with other evidence types to upgrade the confidence level. Current types of evidence for RIs, their classification in groups, and their levels of confidence are summarized in Figure 1. We talk of 7 groups of binding evidence since author statements or inferences by curators are not considered reliable enough, and therefore we exclude them in the confidence assignments. Incidentally, the current confidence level for RIs is limited to their TF site binding evidence, while the evidence for function is a requisite. This is no surprise; certainly, our curation provides evidence supporting genetic elements, but we do not keep track of methods or evidence supporting interactions among objects, plus, only few highly characterized regulatory systems have conclusive evidence supporting which specific promoter is being regulated by which TFRSs.

We assigned each evidence type to one of seven possible groups (Figure 1) and defined the combinations that upgraded the object confidence level using the group numbers. Evidence types in the same group are considered to share methodological bias and cannot be combined to upgrade the confidence levels, while evidence types from different groups are considered independent and their combinations upgrade the object confidence level. Currently, only the evidence types of groups 1 and 2 are classified as strong, groups 4 to 7 are considered weak evidence types (Figure 1). The evidence types 4, 5, and 6 belong to the category HT; the evidence types from group 4 are considered independent from the 5 and 6 types because the methods are considered different enough, with the first group assayed *in vivo* while methods of groups 5 and 6 identify binding *in vitro*; the experimental and computational processing of raw data are also different. DAP-seq and gSELEX are also considered independent of each other. Based on these groupings, the combinations that upgrade that increase the confidence level are the following:

1) Two independent binding evidence types with confidence level “weak” (groups 4–7) upgrade the object confidence level to “strong”.
2) Two independent binding evidence types with confidence level “strong” (groups 1 or 2) upgrade the object confidence level to “confirmed”.
3) Two independent binding evidence types with confidence level “weak” (groups 4–7) in addition to a strong evidence type (group 1 or 2) upgrade the object confidence level to “confirmed”.
4) Four independent binding evidence types with a weak confidence level (groups 4–7) upgrade the object confidence level to “confirmed”.

It is worth mentioning that we applied such increases in confidence provided the evidence for the effect in regulation, or function, is not missing in RegulonDB. As mentioned, binding of purified protein and site mutation are the only evidence types with a strong confidence level. Site mutation is classified as a strong evidence type because it involves the precise identification of the regulatory site, which, if modified, shows no effect on transcription, either *in vivo* (Baseggio et al., 1990; Venkatesh et al., 2010), through a reporter gene or by *in vitro* transcription even in the presence of its TF (Schneiders et al., 2004). Binding of purified protein includes two similar methodologies: electrophoretic mobility shift analysis (EMSA) and footprinting, in which the TF binding to a specific sequence target is probed *in vitro*. Note that currently HT methods are not sufficient to provide a confirmed confidence level, as there are only three independent HT groups of methods, unless another weak evidence is also included. In fact, some HT methods (i.e., ChIP-seq) frequently adds a computational identification of the binding site enhancing its confidence level. There is no single evidence with an assigned level of “confirmed”.

The complete set of evidence combinations that upgrade an RI confidence level can be found under the “regulatory interactions” of the “Stage II. Assignment of confidence level based on additive evidence types” section of the webpage: https://regulondb.ccg.unam.mx/manual/help/evidenceclassification. For instance, ChIP-chip, ChIP-seq, and ChIP-exo belong to group 4, whereas gSELEX belongs to group 5. The rule (4/5/expression)-S means that if an RI has evidence from any method in group 4 plus any evidence from group 5, and any evidence of expression (which supports the functional regulatory evidence) together they upgrade two weak binding evidence types into a strong confidence level.

Once all these updates were in place, we recalculated the confidence levels for two versions of the complete set of RIs present in RegulonDB, i.e., the version before and the one after adding the binding evidence of all current binding HT collections. This is presented in section 2.5 on the “Architecture of Knowledge.” But before we need to explain another implementation that enhances the quality of knowledge representation of RIs in RegulonDB.

### 2.3 Three representations of regulatory interactions: TF-promoter, TF-TU, and TF-gene

A different challenge we have addressed when searching for the best possible way to encode knowledge is the need for intelligent ways to deal with partial knowledge. It is not uncommon for a curator to have to choose the least costly assumption when knowledge is lacking. For instance, years ago, since by definition a TU has a promoter, we added the so-called “phantom promoters” to those TUs that had no characterized promoter. This was eventually eliminated as suggested by Rick Gourse in an EcoCyc meeting, to avoid confusion by users. Another example illustrating the same problem was how to deal with the curation of RIs. Historically, we curated RIs affecting a given promoter, even when there was no such specific evidence. The curator uploaded the RI when the target gene had only one promoter, and if the target gene had two or more promoters, the new RI was mentioned in notes of the TU. It is important to be aware that our curation work has been evolving for more than 30 years now. In this long period, we have added new objects, new features, improved our evidence codes, in addition to many more changes, searching to improve the quality of knowledge representation.

In order to minimize assumptions in our curation process, we defined three levels of description of RIs, which we use depending on the level of detail of knowledge available. We call these “RI types”:

1) The most precise knowledge is when there is evidence that identifies the regulated promoter affected by an RI. Most of these come from classical experiments. In these cases, it is reasonable to deduce that TUs associated with the regulated promoter are regulated by the new RI. These are RIs described at the level of “TF-promoter.”
2) A less detailed description is when the regulated promoter is not known and there is evidence of a change in expression of a group of adjacent genes on the same strand of the promoter that matches with an existing TU with or without promoter. In such cases, we associate the new RI to the existing TU. We call these TF-TU RIs. If there is no previous TU, we create a new TU without a promoter and with evidence of coexpression and link to it the new RI.
3) Finally, when the regulated promoter has not been identified and there is evidence of differentially regulated transcription of the downstream gene(s) from a TF binding site, we create a new RI for which the target is the gene. We call these TF-gene RIs.

As a result, we currently have three means of adding RIs, depending on available knowledge in RegulonDB and EcoCyc: TF-promoter, TF-TU, and TF-gene.

The curation of knowledge related to RIs exerted by a TF depends on several rules. The easy case is when there is no previously annotated RI with the same TF and target gene; in this case, a new RI at the adequate level is annotated, according to the available knowledge. However, if there is a previous RI, of any of the three types, and the new and previous knowledge are consistent, the new evidence is added to the existing RI.

The current RegulonDB version 12.2 portal offers the TF-RI set with labels on the type (TF-promoter, TF-TU, or TF-gene); and it includes the complete list of evidence types. This enables users to exclude specific methods, for instance ChIP-seq evidence when evaluating ChIP-seq data preventing evaluating this new data with previously performed ChIP-seq data. This file is found in the downloadable files (https://regulondb-prerelease.ccg.unam.mx/datasets).

### 2.4 Incorporation of HT-binding evidence to the current collection of RIs

It is important to note that until now, HT-supported RIs have been extracted only when evidence of binding and function are reported in the same publication. However, most studies reporting genome-wide TF binding do not report TF-dependent differential gene expression; in some cases, it is assayed for a small set of TF-binding target genes. Thus, HT datasets contain TFBSs, some of which may support regulation as future studies might show, whereas RegulonDB gathers TFRSs, that is to say, binding sites that support a regulatory interaction. In order to maximize the use of this data we mapped the peaks from the HT-binding datasets to existing RIs in RegulonDB and added such HT-binding evidence to known RIs, increasing their confidence levels.

**FIGURE 2:**
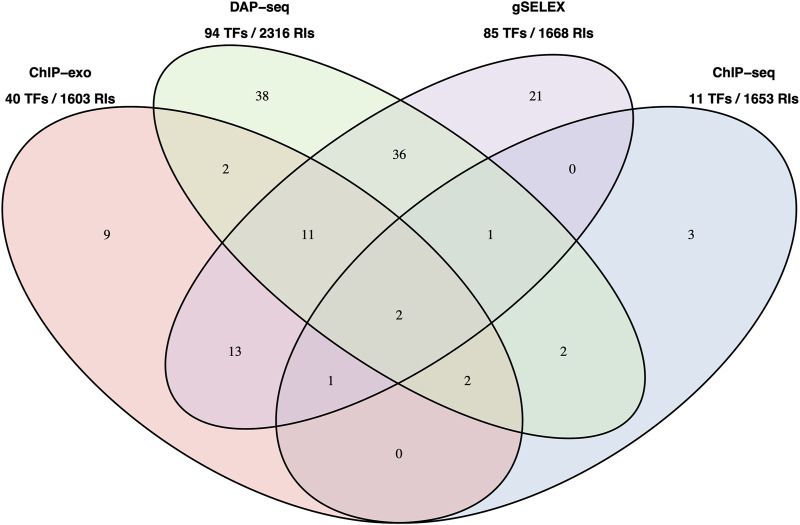
Number of TFs with data in RegulonDB and number of TFBSs available HT datasets. Number of TFs for which we have either dataset for a single or for multiple HT methods. This is the reduced set of TFs for which RegulonDB has at least one regulatory binding site. The TFs names are listed in Supplementary Data Sheet S2. Below the method is the total number of TFs and the number of RIs in RegulonDB for those TFs, that is, the set of candidate RIs that can be matched by each method.

Figure 2 below shows a Venn diagram with the number of TFs with data in RegulonDB currently available for the four HT methodologies, and with the total number of binding sites as reported by authors, and available in our pre-processed datasets. Note that for gSELEX only those datasets with a defined cut off level were mapped.

It is interesting to note that, despite the significant difference in TFs (94 for DAP-seq vs. 11 for ChIP-seq), the total number of candidates TFBSs in these collections that could match RegulonDB RIs is relatively similar.

The addition of HT evidence from the datasets led to an enrichment of binding evidence for 1329 RIs, causing changes in the RI confidence levels, as well as shifts in evidence categories from “nonexperimental” to “HT” and from “classical” to “classical and HT” as discussed below (see Tables 1 and in Supplementary Table S1). Table 1 illustrates, by promoter type and confidence level, the changes in the totals resulting from the addition of HT evidence from the current HT-datasets.

**TABLE 1:** Number of RIs grouped by type and by confidence level.

RI type	Counts (% from total)	Confidence level	# RIs before mapping HT-TFBSs datasets	# RIs after mapping HT-TFBSs datasets
TF-promoter	3954 (72%)	Confirmed	772 (19.5%)	1157 (29.2%)
		Strong	1897 (48.0%)	1710 (43.2%)
		Weak	1285 (32.5%)	1087 (27.5%)
TF-TU	265 (5%)	Confirmed	18 (6.8%)	33 (12.5%)
		Strong	210 (79.2%)	197 (74.3%)
		Weak	37 (14.0%)	35 (13.2%)
TF-gene	1247 (23%)	Confirmed	22 (1.8%)	29 (2.3%)
		Strong	789 (63.3%)	786 (63.0%)
		Weak	436 (35.0%)	432 (34.6%)

The fourth and fifth column show the number of RIs, of each subgroup, i.e., the first row corresponds to the number of TF-promoter RIs, with a confirmed confidence level, 772 before mapping and 1157 after the mapping of HT, datasets. The percent value is relative to the total number of RIs, of the corresponding type, indicated in the second column.

The current public version of RegulonDB RIs contains the results of this mapping of HT evidence types. The current total number of RIs in RegulonDB is 5,466 from 237 different TFs, of which 164 have at least one HT dataset. Additionally, 41 putative TFs have an HT-TFBSs dataset but no RIs in RegulonDB.

In the following 2.5 section we will analyze the architecture of this updated comprehensive collection of RIs currently present in RegulonDB, and in section 2.6 we will focus on evaluating the contribution of HT evidence to the confidence levels of the RIs collection.

### 2.5 Architecture of knowledge supporting the RIs

Given the improved level of detailed updated annotations for all RIs currently available in RegulonDB, we can analyze the contribution of different methods to the current corpus of knowledge of the transcriptional network, at the level of classical vs. HT categories, as well as at the precise level of individual methodologies; their impact in the confidence level of the collection, their most frequent combinations, and for HT methods their recovery of regulatory interactions supported by classical methodologies.

**FIGURE 3:**
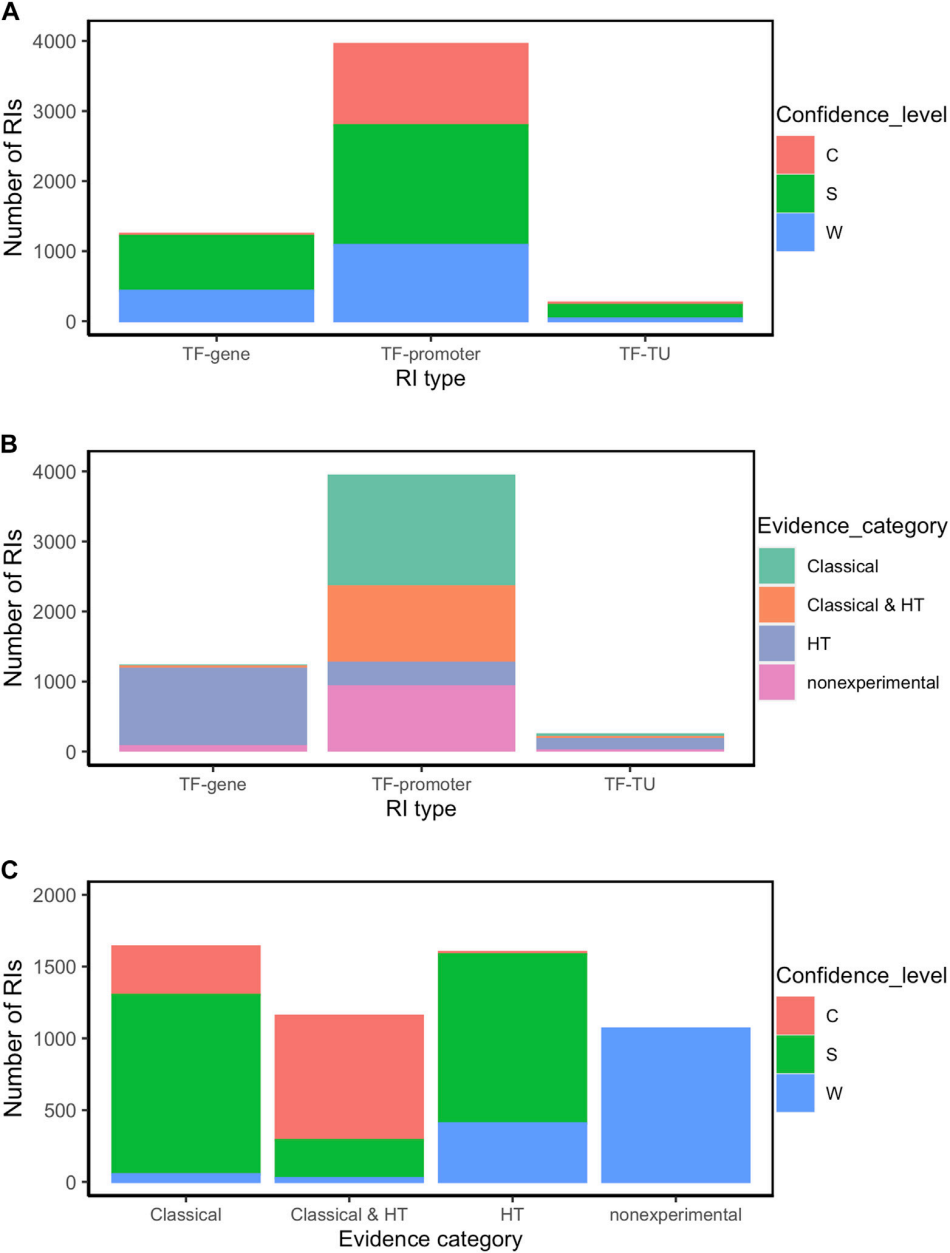
RI distribution analysis by type of RI (TF-promoter, TF-TU or TF-gene), confidence level (C: confirmed, S: strong, and W: weak), and evidence category (classical, HT and nonexperimental) **(A)** Number of RIs by type of RI for each confidence level; **(B)** Number of RIs by evidence category for each type of RI; **(C)** Number of RIs by confidence level for each evidence category. For simplification, RIs were classified within all experimental categories irrespective of whether additionally they also have computational evidence. Thus, for instance, an RI with both computational and classical evidence is counted as “classical”.

RegulonDB version 12.2 contains a total of 5,466 RIs of which 22% are confirmed, 49% strong and 28% have a weak confidence level. The sum of confirmed and strong levels includes over 70% of all current RIs. This indicates the high degree of confidence of the *E. coli* regulatory network, a result achieved thanks to the aggregation of both evidence accumulated by molecular biology through the years, and the more recent HT methods including the mapping process described before (See Figure 3C). In terms of sources of evidence**,** 80.5% have experimental support, with 30% classical, 29% HT and 21% with both classical and HT, whereas 19.5% are nonexperimental**.** In this section we highlight some of the relations between evidence type, category of methods, confidence level, and RI level of description.

The source of knowledge or category of methods is the major determinant of the confidence level. Thus for instance, as mentioned the current collection with all evidence types has 71% of RIs at the higher levels of strong and confirmed evidence. As shown in Figure 3C (see also Supplementary Table S2), among the interactions supported by both classical and HT (1157), the large majority 98% (1137) are either strong or confirmed (Second bar in Figure 3C), with almost the same fraction when counting those with only classical methods (1640 RIs), where the large majority (97%, 1587) are either strong or confirmed (First bar in Figure 3C). These and other observations below show the current relevance of classical methods in supporting reliable (strong and confirmed) evidence. Conversely, the dominant source of weak evidence types come, as expected, from nonexperimental, mostly computational predictions, and some supported by HT methods (Figure 3C).

The tiny fraction of interactions with weak evidence with experimental support come from methods like binding of cellular extracts for instance, as well from those curated years ago when we did not curate the functional evidence of RIs.

In terms of RI type, 72% are of the TF-promoter type, 23% are TF-gene, and only 5% are TF-TU, as shown in Table 1. As expected, the TF-promoter type is the one with the highest number of strong and confirmed interactions (Figure 3A); this is no surprise since, as mentioned before, the TF-promoter level is the one where more mechanistic knowledge of the RI is known.

**FIGURE 4:**
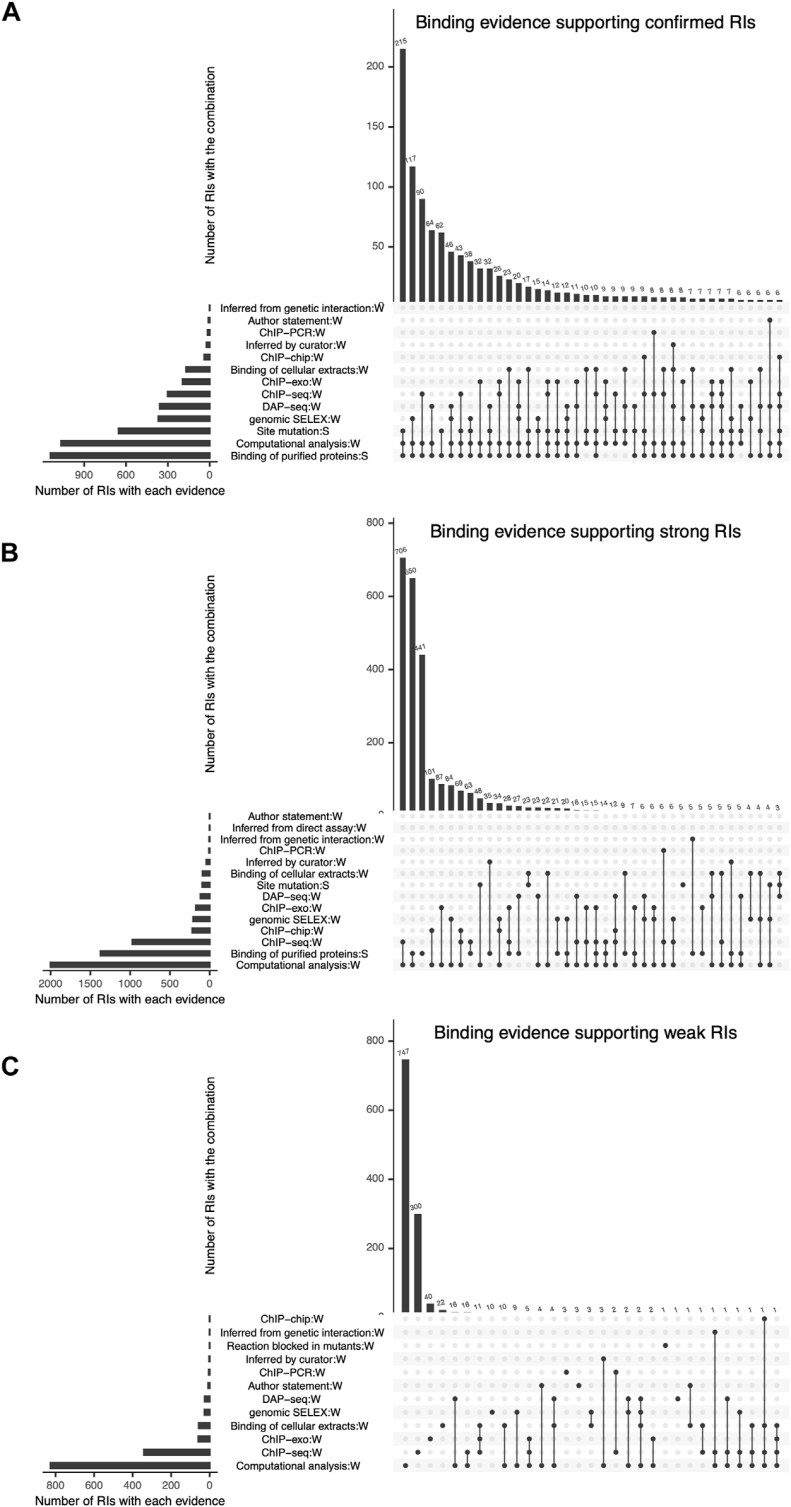
Detailed combinations of binding evidence supporting RIs, shown as intersecting sets using an upset plot. In each plot, the bottom left bars represent the different types of evidence curated and the respective number of RIs they support. The top bars represent the number of RIs supported by each possible combination of 2 or more evidence types. The three plots show the combinations of evidence supporting the current set of **(A)** “Confirmed” RIs, **(B)** “Strong” RIs and **(C)** “Weak” RIs, respectively.

Figure 3A also shows that the highest level of confidence is almost exclusively (95%) dominated by RIs described at the level of TF-promoter, implying that for practically all currently confirmed interactions we know the regulated promoter. When adding both confirmed and strong, this fraction diminishes to 73%, because there is a good number (786) of interactions with strong confidence described at the TF-gene level which come essentially from HT-methods. See in Figure 3B that the TF-gene level gathers mostly HT supported interactions. This matches with the fact -shown in detail in Figure 4B below-that strongly supported interactions currently come mostly from ChIP-seq combined with computational evidence. Nonetheless, it is relevant to keep in mind that close to three-quarters of the current subset combining strong and confirmed evidence have their regulated promoter known. On the other hand, the TF-gene and TF-TU RIs are mainly supported by HT evidence (Figure 3B), and, surprisingly, they have mostly strong confidence levels coming from HT methods (See Figures 3A, B).

It is important to note that not all TF-promoter regulatory interactions come from classical experiments. Currently, 76% have experimental support with 67.6% either classical or classical and HT support, and 8% (333 RIs) only HT; the remaining 24% accounting for 947 interactions are supported only by nonexperimental predictions (See Supplementary Table S1). This explains why 27.5% of TF-gene interactions have a weak confidence level. Note that the categories of methods discussed here refer exclusively to those supporting the TF binding site and its regulatory effect. Their corresponding regulated promoters might in turn be supported by classical, HT or computational predictions. In fact, the promoter and TU methods have also been classified in terms of confidence levels following the same general principles outlined above, but we have not embarked on the combined analyses of evidence types of RIs and their corresponding promoters.

Finally, an interesting consequence of the integration of evidence from three independent groups of HT methods (groups 4, 5 and 6 in Figure 1) is that in principle there can be RIs at the confirmed level supported exclusively by HT plus computational evidence. Figure 3C shows a tiny confirmed orange band in the HT column.

#### 2.5.1 Combination of specific methods and their frequency

The most frequent combination supporting confirmed RIs is “site mutation” (classical evidence) with “binding of purified protein” (classical evidence) and “computational analysis” (nonexperimental evidence) (Figure 4A). It is interesting to consider that these 215 confirmed RIs that contribute with 18% of the total confirmed, would still be confirmed even if we eliminated the computational evidence, since each of the remaining two classical evidence types provide strong support. The second contributor to confirmed RIs is “binding of purified protein” (classical evidence) combined with “genomic SELEX” (HT evidence) and “computational analysis” (nonexperimental evidence) (Figure 4A).

Strong confidence is also strongly dependent on HT and computational analysis support, as six of the seven combinations that contribute most involve these two evidence types in addition to “binding of purified proteins” (classical evidence). (Figure 4B).

**FIGURE 5:**
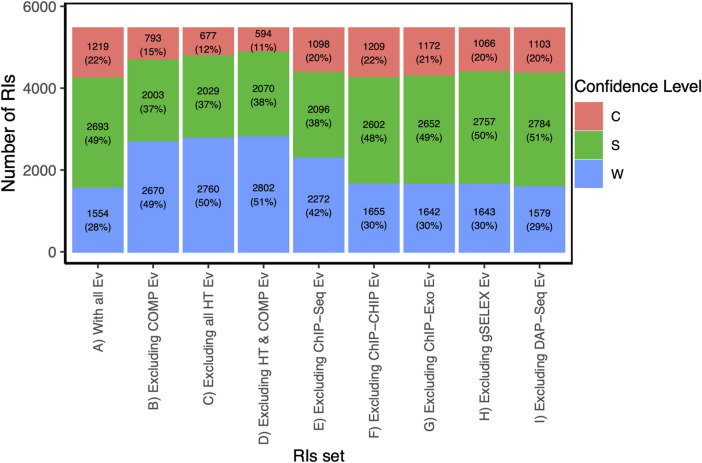
Distributions of confidence level from the RI set when excluding HT and/or computational evidence. Footnote: For the current RI collection, this figure shows the profile in confidence levels as different methods are excluded in order to detect their contribution. The reference is the first column with all evidence types included showing the highest fraction of strong and confirmed levels, and a smaller weak component. For instance, when comparing with bar C, we can quantify how much HT methods are contributing to the confidence levels.

The contribution of computational analyses (group 7 in Figure 1) to the strong and confirmed levels raises the question of how adequate it is to consider it independent from the experimental methods. We know that there is no *ab initio* bioinformatics, since all computational analyses start with data coming from experimental work, in the form of subsets of either binding sites, regions or peaks from ChIP methods, or upstream regions from co-regulated genes, for instance. On the other hand, it is true that identifying sites that match to a motif that fits features of known TF motifs (i.e., size, information content), contributes to the confidence of the results. The specific combinations in Figures 4A, B can be used to estimate the impact in confidence when eliminating the computational evidence. Using our Confidence Level Calculator Tool publicly available (See Methods), we generated the fraction of strong and weak confidence when the computational analysis is eliminated. The profile changes to 15% confirmed, 37% strong and 49% weak; that is to say half, 52%, of interactions belong to the two strongest categories, considerably lower than the 71% in the current collection when all evidence types are included (Compare first and second bars in Figure 5).

Most of the weak RIs are supported only by “computational analysis” or by “ChIP-seq” evidence types (Figure 4C). As mentioned before, HT evidence is considered weak, so RIs supported by only ChIP-seq have a weak confidence level (Figure 4C). There are also some weak RIs supported by classical evidence types, as already mentioned. In Figure 4C, we can observe that some RIs are supported by different combinations of independent evidence types and they do not become strong, this is because for these RIs the evidence of function (effect over expression) is missing, probably due to the historic process of curation. Future curation will enable us to recover their functional evidence. Note that 100% of nonexperimental RIs are classified as weak (Figure 3C).

### 2.6 How HT evidence is changing the landscape of knowledge

In terms of confidence levels, currently, RegulonDB (version 12.2) contains a total of 5,466 RIs, of which 22% have confirmed evidence, 49% have strong evidence, and 28% have weak support (Table 1 fifth column; Figure 5 bar A). The sum of confirmed and strong levels includes over 71% of all current RIs. As shown in Figure 5 in the absence of HT evidence the numbers of high confidence drops significantly, with 12% confirmed, 37% strong and barely more than half, 50.5%, weak confidence. Does this mean that close to 20% of the strong plus confirmed come from HT support alone? Let us look first at one specific example.

The RI Cra-frubp involving the site 2263516–2263533, has a weak confidence level when ignoring all HT evidence since it is only supported by computational evidence and by gene expression analysis. However, the binding covering this site is also supported by ChIP-exo, gSELEX, and DAP-seq. These independent HT evidence types together with computational evidence upgrade the RI confidence level to confirmed. A total of 16 sites have a confirmed level thanks to HT experiments, otherwise they would be weak. See the tiny orange band in Figure 3C on top of the HT column. This specific example illustrates a general observation: The strong effect of HT methods in improving confidence level happens thanks to their association with computational evidence.

A careful comparison of the first 4 bars in Figure 5 shows, first, that eliminating either the computational or the HT methods affect quantitatively very similar the overall confidence counting; and eliminating both does not change the effect. Thus, it is clear that it is their combined presence that has such a strong effect in the overall profile. The specific dominant combinations of methods for each confidence level shown in Figure 4 panels A and B also help us to see that the confirmed level still strongly depends on classical methods, as opposed to the strong category. Also notice the particular relevance of ChIP-seq within the HT methods.

In terms of the global categories “classical”, “HT”, “classical and HT” and “nonexperimental”, there are 1640, 1601, 1157 and 1068 RIs, respectively. RIs with classical evidence (classical + classical and HT) represent 51.2% (2797). We are still at a time where classical methods contribute significantly. This is expected to change in the near future as global genomic methods dominate the landscape, provided these global strategies provide both binding as well as functional evidence supporting the regulatory effect.

### 2.7 Recovery of classical TF binding regulatory sites by HT methods

As mentioned, the results of HT methodologies are frequently compared with the RegulonDB data as a way to validate them. However, these analyses had been performed with the complete set of RIs, maybe users ignoring the fact that this set includes RIs supported by HT methods. Thanks to the multiple improvements presented in this work, we can now use specific gold standard datasets that exclude specific sources, to avoid circularity.

**FIGURE 6:**
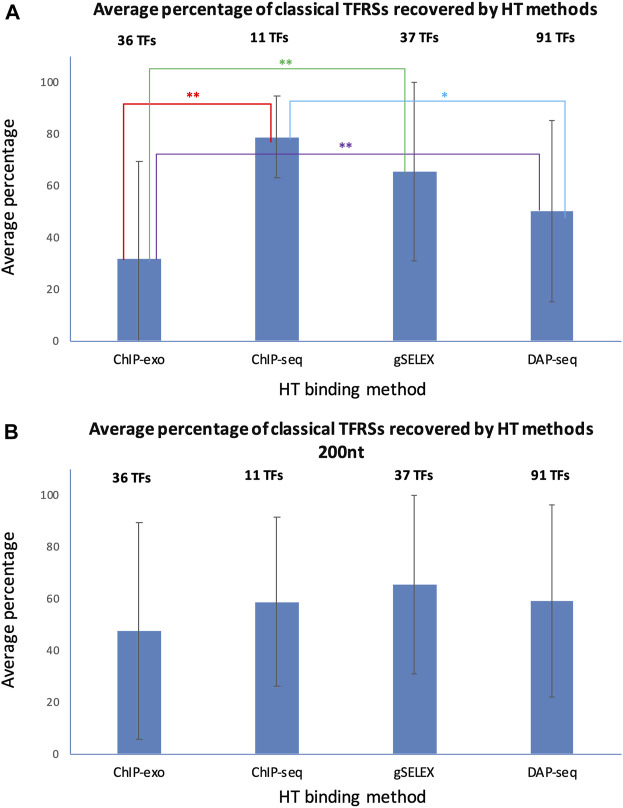
Recovery average of classical TFRSs by different HT-binding methodologies. For each methodology, the fraction of recovered TFRS sites in RegulonDB was estimated and the average for all TFs for each method and std deviation is shown. The panel **(A)** shows the results using variable peak sizes based on data as reported by authors, and panel **(B)** shows the results using a peak size of 200 for all HT TF-binding datasets. The set of TFs is specific to each method given the currently available datasets gathered in RegulonDB version 12.2 and also limited to those TFs for which there is at least one classical TFRS in RegulonDB (For data details see Suppementary Material S2). For the statistical test see Methods. Two stars indicate statistically significant differences, as mentioned in the main text.

To assess the performance of HT-binding methodologies in recovering sites from classical RIs in RegulonDB, we considered only the subset of TFs that have at least one classical TFRS. For each TF we calculated the percentage of classical TFRSs that map with the peaks in the corresponding dataset, the average percentage was calculated for the subset of TFs available for each HT methodology. In Figure 6 below we show the recovery of classical TFRSs. ChIP-seq was the methodology that recovered the highest percentage (78.7 ± 15.8%), followed by gSELEX (65.4 ± 34.4%), DAP-seq (51.0 ± 35.2%) and ChIP-exo (31.6 ± 37.8%) (See Supplementary Data Sheet S3). Statistically significant differences (*p*-value < 0.05) were observed between ChIP-seq and ChIP-exo (*p* = 0.0052); ChIP-exo and gSELEX (*p* = 0.0024); and ChIP-exo and DAP-seq (*p* = 0.342) (indicated by two stars in Figure 6). Additionally, the dissimilarities between ChIP-seq and DAP-seq (*p* = 0.0632) approached significance (one star in Figure 6). A different form of comparison is to calculate the percentage of the total number of classical RIs mapped for the subset of corresponding TFs. Using this approximation, similar results are obtained with 72.6% of total classical TFRSs recovered by ChIP-seq, 50.3% recovered by gSELEX, 28.3% recovered by DAP-seq and 22.5% recovered by ChIP-exo (See Supplementary Data Sheet S3).

The mapping in Figure 6A shows the results when considering, as much as possible, the peak size reported by the authors. This involved taking into account both the reported start and end of the peak. When only the center of the peak was reported, a peak size of 200 was used. The gSELEX datasets only contain peak center information, so a peak size of 200 nt was used in both comparisons. For ChIP-seq, 6 datasets contain peak centers, while the rest show high variability in peak sizes, even within the same datasets. The average peak size was 582 nt (with a standard deviation between datasets of 608 nt). For DAP-seq, a peak size of 55 was found (with a standard deviation between datasets of 4.6 nt), and finally, for ChIP-exo, a peak size of 34 nt was identified (with a standard deviation between datasets of 14 nt).

Considering these results, we wondered to what extent the peak size impacts the classical TFRSs recovery for each methodology. We repeated the mapping with a peak size of 200 nt for all datasets, with the peak center as the reference point. The results in Figure 6B and in Supplementary Data Sheet S4 show a decrease in the recovery by ChIP-seq, and an increase by DAP-seq and ChIP-exo, as expected. In this case, we found no significant differences among the methods. This mapping comparison shows, as expected, the relevance of peak size on the results. Subsequently, the same analysis was performed using the dataset of TFRSs from RIs with a confirmed confidence level. As shown in Supplementary Figure S2, similar results were obtained. Once again, when using peak sizes as reported, ChIP-seq was the methodology that recovered the highest average percentage (94.0% ± 8.7%) of classical confirmed RIs, followed by gSELEX (76.4% ± 35.4%), DAP-seq (57.3% ± 39.6%) and ChIP-exo (36.1% ± 39.3%) (Supplementary Figure S2A, Supplementary Data Sheet S5). The numbers are again modified when using the same 200 bp peak length (Supplementary Figure S2B, Supplementary Data Sheet S6).

In summary, ChIP-seq recovered the highest fraction of sites supported by classical methods but no statistical significance was found in comparison with gSELEX or DAP-seq. ChIP-exo shows the lowest performance of all methods, but with a much higher precision in binding sites. The high recovery by ChIP-seq is interesting since classical methods are by far *in vitro* experiments such as the gSELEX and DAP-seq as opposed to the ChIP methods where binding is identified *in vivo*. These preliminary results suggest that, under the conditions performed, there is no major difference in the *in vivo* vs. *in vitro* specific binding of TFs in the *E. coli* genome.

These comparisons should be clearly taken with a grain of salt given the limited datasets and their heterogeneity. To start with, as shown the largest peak sizes of ChIP-seq contributes to its higher recovery, and although ChIP-exo recovery is smaller, its precision in locating specific binding sites is considerably better as indicated by its small average peak size. Also, remember that the subset of TFs here analyzed for each method is different as shown in Figure 2. Furthermore, all ChIP-exo experiments come from a single (Palsson´s) research team, similar to the gSELEX (Ishihama´s) experiments and the DAP-seq collection from a single publication (17)**,** whereas the ChIP-seq collection comes from diverse authors. In the papers of the 11 TFs with ChIP-seq data it was not uncommon to find experiments performed with different supplements -potential allosteric metabolites involved in TF binding-, and the datasets we gathered include all those different TFBSs found. Our curation role is to gather what authors publish, implying for instance, that we respected the length of peaks when offered by the authors; whenever the dataset included only the peak center we arbitrarily defined peak lengths of 200 bp. Thus, there is clearly room for confounding factors limiting the interpretation of these quantitative comparisons.

As already observed, see Figure 2, when counting the total set of RIs for potential matching for each method, their number is not as different as one could expect given the considerably larger difference in number of TFs. This is consistent with the fact that the TF network connectivity follows a power-law, and in this case it contributes to a more even number of sites per method that could match with a classic RI site. Another factor is that ChIP-seq experiments, as mentioned, frequently benefit from various experiments performed with various supplements for the same TF increasing the chances to recover more classical TFRSs.

### 2.8 Use of gold standard datasets to benchmark HT-binding methodologies

The efforts invested in curating evidence for RIs using specific codes for different methodologies, along with their classifications into independent groups, confidence levels, and categories, now enable us to filter and create subsets of RIs. These subsets can serve as a gold standard for benchmarking HT-binding methodologies. The complete set of RIs is available on the RegulonDB website under “Releases and Downloads/Downloads/Experimental Datasets/TF-RISet”. On this page, users can download the entire set, and use two tools available:

1) Browse and Filter: In this tool, filters can be applied to each column to obtain a subset of RIs, and users can download them accordingly. For example, RIs with a confirmed confidence level could be filtered.
2) Confidence Level Calculator Tool: In this tool, one or multiple evidence codes can be excluded, and the confidence level can be recalculated.

With these tools, users can conduct their own analyses.

## 3 Discussion

One relevant outcome of this work is the availability of gold standard datasets useful for benchmarking new methodologies. From the master RI complete table (“Releases and Downloads/Downloads/Experimental Datasets/TF-RISet”) containing all the evidence types for each RI, users can make their own combinations defining confidence levels differently as we did. Users can include or exclude specific subcollections based on the method and/or evidence types and can also select subsets of RIs filtering by confidence levels with the new tools publicly available.

As mentioned, our strategy of confidence levels follows the ideas outlined in the 2013 paper; the major additions presented in this work are the following: we have increased the detail of evidence codes, and therefore, we can now distinguish the contribution of specific methods, such as mutation vs. binding of purified protein, or the different HT methods. We added new evidence codes to include novel HT methods (i.e., ChIP-exo and DAP-seq) and clearly defined 7 groups of independent binding methods. Furthermore, we organized the computational implementation of the whole process that calculates confidence levels based on all or specific subsets of evidence groups (Confidence Level Calculator Tool), and finally, all the process is now documented and publicly available.

Additionally, we have implemented three levels of RIs adequate to capture the diverse cases of partial knowledge. These advances enable the analyses performed of the different sources of knowledge and their contribution to the currently known *E. coli* collection of regulatory interactions. We have analyzed how confidence levels of the RI set increased thanks to the combined HT and computational evidence types in spite of the reduced number of TFs studied so far by genomic strategies. This shows how in the near future HT methods will most likely become the dominant sources of knowledge that will eventually support the whole *E. coli* regulatory network. The RegulonDB model with the current computational and encoding infrastructure here reported provides the foundation to address novel questions on the sources and confidence levels of the corpus of knowledge, as illustrated by the analyses and discoveries we presented.

It might be obvious that the patterns and combinations of methods presented here are a result of experimental practices and should not be interpreted as a measure of congruence across the different methods. This is even more the case given the heterogeneous collection of TFs studied by the different HT methodologies and the many differences already mentioned before, (see section on the recovery by HT methods). A plausible interesting observation of these comparisons is that there are no significant differences in the *in vivo* binding (ChIP methods) vs. *in vitro* binding (gSELEX and DAP-seq) of TFs. Remember that classical methods are mostly *in vitro* binding.

Even if the patterns and combinations of methods will be changing in the future, we see value in this work as: i) We performed a detailed analysis of the sources of knowledge of the current collection; ii) Users can better appreciate the complexity and diversity of sources of knowledge and decide according to their needs what subsets of data they want to work with; iii) We are prepared with all the modifications involved, to keep updating these distributions in future releases and to adequately enrich our collection of tools as new methods and their associated data become available in the future; iv) The analyses bring up gaps in annotations and help us to identify inconsistencies contributing to improve our curation and knowledge representation; and last but not least, v) All the computational processing involved provides a solid foundation for future work to further integrate classic and HT data beyond regulatory interactions, to include in the future transcription start sites and transcription unit datasets. Furthermore, the uniformization to current genomic coordinates, and normalization of data provides the foundation to the comparability of results coming from different HT methods, and the evaluation of their performance in recovery of classical data.

Gold standard datasets are useful for different fields of biomedical research, but they must be not only a reference collection but also one that represents data with the highest level of confidence. The evaluation of data confidence based on independent evidence is a common practice in research; for example, quantitative reverse transcription PCR (RT-qPCR) is used to validate RNA-seq experiments. However, only a few studies have used this approximation to evaluate data on a large scale (Myers et al., 2013). In medicine, levels of evidence are assigned to studies based on diverse criteria, such as quality, with higher levels of quality of evidence entailing less risk of bias (Burns et al., 2011). Our approach can in principle be applied to analyze data from curated databases which have structured evidence codes associated with objects, such as BioGRID database (Oughtred et al., 2021), as well as other molecular databases.

The different improvements discussed in this paper enable us to incorporate HT-generated knowledge together with classical molecular biology methods, since it is easy to dissect subsets based on their supporting methods. The next step will be to add an additional layer based on a similar integrative analysis focused on combining classical and HT methods for TSSs and TUs and update what constitutes one of the best-studied transcriptional regulatory networks of any genome model organism. This is also likely the best computationally represented corpus of knowledge of gene regulation.

## 4 Methods

### 4.1 Updates in RegulonDB

#### 4.1.1 Evidence updates

In RegulonDB version 12.2, we made important evidence-related changes, including: 1) Evidence code. The evidence codes were made more informative, i.e., BPP was changed to EXP-IDA-BINDING-OF-PURIFIED-PROTEINS. 2) Evidence confidence levels. Evidence types were classified as “weak” or “strong” depending on whether they provided physical and direct proof of the existence of the object or interaction.

#### 4.1.2 Object confidence level update

The confidence level for each RI, promoter, and transcription units was calculated and updated using the linked evidence and the additive evidence. The confidence level assignment to RIs is described below; we followed the same principles for the other objects. These changes are contained in the current RegulonDB including the downloadable datasets.

### 4.2 Data source for RIs

All the work presented here was done using RegulonDB version 12.2 synchronized with Ecocyc version 27.0. In this version, the downloadable text file for Regulatory Interactions was made available and also the evidence catalog file. The formats and descriptions of these files are available at https://github.com/regulondbunam/download-data-files.

### 4.3 Confidence level assignments to evidence types and to RIs

The confidence levels were assigned to RIs by a process involving two steps:

Stage I) Each single evidence type was classified into weak or strong, as described in section 2.2.
Stage II) Assignments of confidence levels to RIs were based on the process using the rules for “additive evidence”.

The concept of “additive evidence” was called “cross-validation.” As we proposed a while ago, Weiss et al. (2013) (Weiss et al., 2013), the confidence level of a biological entity depends on the combined evidence derived from mutually independent methods.

We grouped methods that could have similar sources of false positives. This resulted in seven independent evidence groups (Figure 1). The combinations of evidence groups that upgraded the RI confidence levels were defined based on the four rules mentioned in Section 2. We call these combinations additive evidence, which define the final level of confidence assigned to each RI. The complete set of group combinations that upgraded RI confidence levels can be found under the “regulatory interactions” of the “Stage II” section of the webpage: https://regulondb.ccg.unam.mx/manual/help/evidenceclassification.

### 4.4 Access options for users

Although it is well known that RegulonDB contains a comprehensive collection of experiments performed through decades of classic methodologies (to the extent that the literature has been found by our work in collaboration with EcoCyc), users must be aware that we have already incorporated evidence from HT methods.

The current publicly available RegulonDB offers downloadable datasets grouping collections of objects in https://regulondb.ccg.unam.mx/datasets. The first option offers the “RIset” which contains all the evidence types for binding and function of RIs, in columns 21 and 22 respectively. These can be used to filter and extract, for instance, the subcollection supported only by classic methods. The same strategy could be used to select RIs supported by a specific evidence type. Users can also subselect RIs based on the confidence level, or on the different groups of methods, as described in Figure 1. Furthermore, users may define their own rules and categories of different levels of confidence and use the whole collection of evidence types to classify each individual RI in a new classification of confidence levels.

All scripts and computational processes built to generate the data and analyses presented in this paper are publicly available and can be found at https://github.com/PGC-CCG/supplementary-material/tree/master/gold-standard.

### 4.5 Analysis of the current set of RIs

The analyses of the anatomy of RI knowledge presented here were performed using R (2022.06.23, version 4.2.1), Rstudio (2022.07.1, Build 554), and the ggplot2 (version 3.4.0) library.

### 4.6 Mapping RIs from RegulonDB to the collection of HT TF-binding datasets

The collection of HT TF-binding datasets contains four subcollections with different number of TFs: DAP-seq (107 TFs), ChIP-seq (11 TFs), ChIP-exo (73 TFs), and gSELEX (121 TFs). The mapping of RIs from RegulonDB to the HT TF-binding datasets to enrich the RIs evidence involved the subset of TFs that have both sites in RegulonDB and datasets with defined cut off levels subcollections resulting in: 94 TFs for DAP-seq, 11 for ChIP-seq, 40 for ChIP-exo and 85 for gSELEX (See Supplementary Data Sheet S2).

In order to make these collections comparable among them and with RegulonDB TFRSs, multiple steps were implemented that together constituted what we call “mapping,” of RIs with HT-binding data. This mapping involves:

#### 4.6.1 Uniformization of the genome coordinates for all datasets

The coordinates of the DAP-seq datasets were published using the last genome version of the *E. coli* str. K-12 substr. MG1655 (U00096.3), so they were not modified. The ChIP-seq, gSELEX, and ChIP-exo datasets with coordinates in the past genome version (U00096.2) were updated to version U00096.3. The corresponding Scripts are found in the github indicated.

#### 4.6.2 To map the RegulonDB RI set with peaks from the HT-TFBSs subcollections

A program in Python was implemented to compare each RI binding site with each peak corresponding to the same TF. A match is assumed when the RI site coordinates are both within the region covered by the HT peak. When authors report the two coordinates of a peak, we keep using such sequences; when authors report only the peak center, we extract 100 bp on each side making a peak of 200 bp.

When a match between a site from a RI and the HT binding-peak is found, the evidence of the corresponding HT-methods is added to the corresponding RI. This process is executed in each RegulonDB release. Scripts are found in github as mentioned.

### 4.7 HT-binding methodology efficacies in recovering sites from classical RIs in RegulonDB

To evaluate the performance of HT-binding methodologies (ChIP-seq, ChIP-exo, gSELEX, and DAP-seq) in recovering Transcription Factor Regulatory Sites (TFRSs) from RegulonDB, the RISet was filtered to exclude circularity, as detailed below. For each methodology, TFs were evaluated if they had at least one dataset in the subcollection of HT TF-binding and at least one RI in the filtered set from RegulonDB. The filtered RISet was mapped to the datasets from each methodology as mentioned before. Afterwards, the total number of sites in the subset of RIs mapped and the percentage of them detected for the corresponding methodology were calculated for each TF. Finally, the average percentage of sites recovered was calculated for each methodology. For the analysis of gSELEX, only the datasets with a cutoff defined by authors were mapped. This means that datasets with a 0% cutoff and those that include the 40 peaks with the highest binding intensity were excluded.

#### 4.7.1 RISet filtering

The Regulatory Interaction Set (RISet) from RegulonDB was filtered to generate two different subsets used for comparisons:

1) The classical TF RISet, consisting of RIs with at least one classical binding evidence. It was generated using the “Browse and Filter” tool from RegulonDB, as described earlier.
2) The classical confirmed TF RISet generated using the “Confidence Level Calculator” tool. Initially, the confidence level of RIs was recalculated, excluding all HT-binding evidence types. Subsequently, the RIs were filtered based on confirmed confidence level and having at least one classical binding evidence.

#### 4.7.2 Peak size analysis

The average peak size for each dataset and for each subcollection of HT TF-binding was calculated with a Python program. This calculation was performed only for datasets that specify the peak start and the peak end.

#### 4.7.3 Impact of peak size

To understand the influence of peak size on TFRS recovery, a comprehensive analysis was carried out. The mapping of the filtered RISet was repeated, this time using a uniform peak size of 200 nucleotides for all datasets. This allowed a comparative assessment of recovery percentages across methodologies.

#### 4.7.4 Statistical analysis

To evaluate differences in the detection performance of the methodologies we conducted a Kruskal–Wallis test, along with the Bonferroni correction, to compare mean rank differences across the four groups, considering the non-parametric nature of the data and the variability in the number of TFs analyzed in each case.

### 4.8 Resource Identification Initiative

To take part in the Resource Identification Initiative, please use the corresponding catalog number and RRID in your current manuscript. For more information about the project and for steps on how to search for an RRID, please click here.

## Data Availability

Publicly available datasets were analyzed in this study. This data can be found here: https://regulondb.ccg.unam.mx/datasets https://regulondb.ccg.unam.mx/ht/dataset/TFBINDING/.
